# Supplementary material for: Chronic oxytocin administration stimulates the oxytocinergic system in children with autism
Source: Nat Commun. 2024 Jan 2;15:58. doi: 10.1038/s41467-023-44334-4 (PMC10762037; doi:10.1038/s41467-023-44334-4)
Supplement: Supplementary file 1 — Supplementary Material [file 41467_2023_44334_MOESM1_ESM.pdf]

Supplementary Material

Chronic oxytocin administration stimulates the oxytocinergic system in children with autism

Matthijs Moerkerke*[1,2], Nicky Daniels*[2,3], Laura Tibermont[1,2], Tiffany Tang[1,2], Margaux Evenepoel[2,3], Stephanie Van der Donck[1,2], Edward Debbaut[1,2], Jellina Prinsen[2,3], Viktoria Chubar[4], Stephan Claes[4], Bart Vanaudenaerde[5], Lynn Willems[5], Jean Steyaert[1,2], Bart Boets[†,1,2], Kaat Alaerts[†,2,3]

[1] Center for Developmental Psychiatry, Department of Neurosciences, KU Leuven, Leuven, Belgium
[2]Leuven Autism Research (LAuRes), KU Leuven, Leuven, Belgium
[3] Research Group for Neurorehabilitation, Department of Rehabilitation Sciences, KU Leuven, Leuven, Belgium

[4] University Psychiatric Centre, KU Leuven, Leuven, Belgium

[5] Laboratory of Respiratory Diseases and Thoracic Surgery, Department of Chronic Illness and Metabolism, KU Leuven, Leuven, Belgium

* These authors contributed equally
[†] These authors jointly supervised this work

Behavioural measures

Table S1. Detailed description of the adopted behavioural measures.

Outcome measures	Construct	Type of report	Meaning of higher scores	Reference
**Social Responsiveness Scale-Children (SRS-2)**	Symptom severity	Parent-reported questionnaire	Greater deficits in social responsiveness	Constantino & Gruber, 2012 [1]; Roeyers et al., 2015 [2]
**Autism Diagnostic Observation Schedule (ADOS-2)**	Symptom severity	Observation	More severe symptoms of autism spectrum disorder	Lord et al., 2012 [3]
**Wechsler Intelligence Scale for Children (WISC-V-NL)**	Verbal Intelligence Quotient	Observation	Higher verbal abilities	Wechsler, 2018 [4]
	Performance Intelligence Quotient		Higher visual spatial abilities	
**Screen for Child Anxiety Related Emotional Disorders (SCARED-NL)**	Anxiety	Parent-reported questionnaire	Higher risk for anxiety disorders	Muris et al., 2007 [5]
**Attachment Style Classification Questionnaire (ASCQ)**<br>**Anxious**<br>**Avoidant**<br>**Secure**	Attachment	Self-reported questionnaire	More anxious, avoidant or secure attachment toward their peers	Finzi et al., 2000 [6]

Supplementary result

Figure S1. Effect of chronic oxytocin administration on *OXTR* DNAm levels at CpG sites -914 and -934. Visualisation of the salivary *OXTR* DNAm levels at CpG -914 and -934 for each nasal spray group (oxytocin and placebo) at each assessment session (T0: $n_{oxytocin}$ = 38, $n_{placebo}$ = 39; T1: $n_{oxytocin}$ = 37, $n_{placebo}$ = 38; T2: $n_{oxytocin}$ = 38, $n_{placebo}$ = 37). Data are presented as mean values, vertical bars denote standard errors.

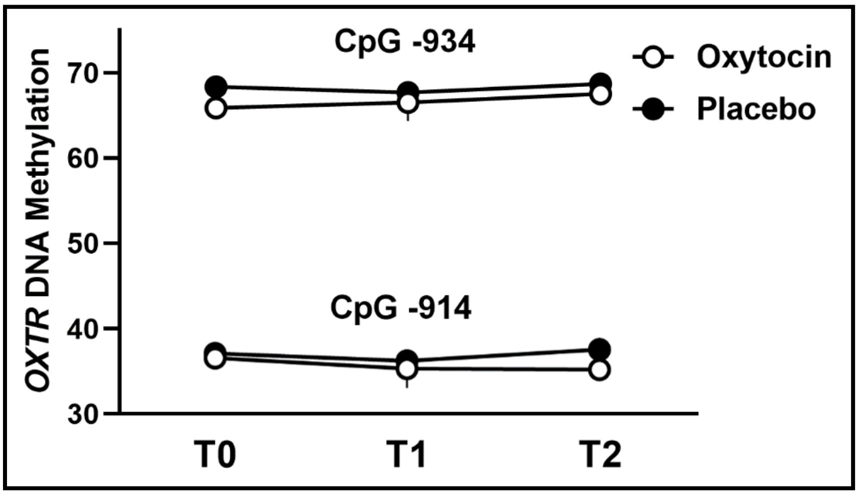

Supplementary references

1. Constantino, J. & Gruber, C. *Social responsiveness scale 2nd. ed: SRS-2. Manual. Western Psychological Services.* (2012).

2. Roeyers, H., Thys, M., Druart, C., de Schryver, M. & Schittekatte, M. Nederlandse bewerking van de SRS-2. *Amsterdam: Hogrefe Uitgevers.* (2015).

3. Lord, C. *et al.* ADOS-Autisme diagnostisch observatieschema Handleiding. (2012).

4. Wechsler, D. WISC-V-NL. Wechsler Intelligence Scale for Children, Fifth Edition, Dutch version. Preprint at (2018).

5. Muris, P., Bodden, D., Hale, W., Birmaher, B. & Mayer, B. SCARED-NL. Vragenlijst over angst en bang-zijn bij kinderen en adolescenten. Handleiding bij de gereviseerde Nederlandse versie van de Screen for Child Anxiety Related Emotional Disorders. Preprint at (2007).

6. Finzi, R., Cohen, O., Sapir, Y. & Weizman, A. Attachment Styles in Maltreated Children: A Comparative Study. *Child Psychiatry and Human Development 2000 31:2* **31**, 113–128 (2000).
