# Supplementary material for: Chronic oxytocin administration stimulates the oxytocinergic system in children with autism
Source: Nat Commun. 2024 Jan 2;15:58. doi: 10.1038/s41467-023-44334-4 (PMC10762037; doi:10.1038/s41467-023-44334-4)
Supplement: Supplementary file 3 — Reporting Summary [file 41467_2023_44334_MOESM3_ESM.pdf]

nature portfolio

Corresponding author(s): Kaat Alaerts

Last updated by author(s): Nov 21, 2023

Reporting Summary

Nature Portfolio wishes to improve the reproducibility of the work that we publish. This form provides structure for consistency and transparency in reporting. For further information on Nature Portfolio policies, see our Editorial Policies and the Editorial Policy Checklist.

Statistics

For all statistical analyses, confirm that the following items are present in the figure legend, table legend, main text, or Methods section.

n/a	Confirmed	
☐	☒	The exact sample size (*n*) for each experimental group/condition, given as a discrete number and unit of measurement
☐	☒	A statement on whether measurements were taken from distinct samples or whether the same sample was measured repeatedly
☐	☒	The statistical test(s) used AND whether they are one- or two-sided<br>*Only common tests should be described solely by name; describe more complex techniques in the Methods section.*
☐	☒	A description of all covariates tested
☐	☒	A description of any assumptions or corrections, such as tests of normality and adjustment for multiple comparisons
☐	☒	A full description of the statistical parameters including central tendency (e.g. means) or other basic estimates (e.g. regression coefficient) AND variation (e.g. standard deviation) or associated estimates of uncertainty (e.g. confidence intervals)
☐	☒	For null hypothesis testing, the test statistic (e.g. *F*, *t*, *r*) with confidence intervals, effect sizes, degrees of freedom and *P* value noted<br>*Give P values as exact values whenever suitable.*
☒	☐	For Bayesian analysis, information on the choice of priors and Markov chain Monte Carlo settings
☒	☐	For hierarchical and complex designs, identification of the appropriate level for tests and full reporting of outcomes
☒	☐	Estimates of effect sizes (e.g. Cohen's *d*, Pearson's *r*), indicating how they were calculated

Our web collection on statistics for biologists contains articles on many of the points above.

Software and code

Policy information about availability of computer code

Data collection	All statistical analyses were executed with Statistica version 14 (Tibco Software Inc.).
Data analysis	All statistical analyses were executed with Statistica version 14 (Tibco Software Inc.).

For manuscripts utilizing custom algorithms or software that are central to the research but not yet described in published literature, software must be made available to editors and reviewers. We strongly encourage code deposition in a community repository (e.g. GitHub). See the Nature Portfolio guidelines for submitting code & software for further information.

Data

Policy information about availability of data

All manuscripts must include a data availability statement. This statement should provide the following information, where applicable:

- Accession codes, unique identifiers, or web links for publicly available datasets
- A description of any restrictions on data availability
- For clinical datasets or third party data, please ensure that the statement adheres to our policy

Data will be made available upon reasonable request.

Research involving human participants, their data, or biological material

Policy information about studies with human participants or human data. See also policy information about sex, gender (identity/presentation), and sexual orientation and race, ethnicity and racism.

Reporting on sex and gender	There is a predominant sampling from boys with ASD: 80%, reflecting the gender ratio in the general population – see Table 1, outlining the demographics of our sample. We stated in the future directions of the discussion that it should be explored whether the observed effects will generalize in samples with a larger representation of girls.
Reporting on race, ethnicity, or other socially relevant groupings	na
Population characteristics	This included symptom characteristics (Autism Diagnostic Observation Schedule, ADOS-2, (Lord et al., 2012); Social Responsiveness Scale-Children, SRS-2, (Constantino & Gruber, 2012)), intelligence quotients (IQ; WISC-V-NL, (Wechsler, 2018)), biological sex, handedness and age, see Table 1, outlining the demographics of our sample.
Recruitment	Children with a formal diagnosis of ASD were recruited through the Leuven Autism Expertise Centre at the Leuven University Hospital (Belgium) between July 2019 and January 2021. There were no self-selection or other biases that may have impacted the results.
Ethics oversight	All study procedures and consent forms were approved by the local Ethics Committee at the KU Leuven (S61358), the European Clinical Trial Registry (EudraCT 2018-000769-35) and the Belgian Federal Agency for Medicines and Health products in accordance with Declaration of Helsinki. Parents/legal guardians of the participants provided informed consents and participants provided informed assents, prior to the start of the study.

Note that full information on the approval of the study protocol must also be provided in the manuscript.

Field-specific reporting

Please select the one below that is the best fit for your research. If you are not sure, read the appropriate sections before making your selection.

☒ Life sciences ☐ Behavioural & social sciences ☐ Ecological, evolutionary & environmental sciences

For a reference copy of the document with all sections, see nature.com/documents/nr-reporting-summary-flat.pdf

Life sciences study design

All studies must disclose on these points even when the disclosure is negative.

Sample size	A total of 80 participants (40 in each treatment arm) participated in the trial, allowing to detect a medium effect size (d = 0.60) with α = 0.05 and 80% power, corresponding to effect sizes previously reported in a four-week oxytocin trial with school-aged children
Data exclusions	for some participants, saliva samples were missing at one or more assessment sessions due to participant being unable or forgetful to bring the sample or due to discontinuation of the study by the participant (see Fig. 1 for the CONSORT flow diagram visualizing the number of included participants).
Replication	Questionnaires and salivary samples were collected and analyzed at baseline (T0), 4 weeks post-treatment (T1) and at 4-week follow-up (T2), in line with prior research (e.g. Alaerts et al., 2021). Specifically, oxytocin levels were assessed via saliva samples acquired at each assessment session (T0, T1, T2), at two time points: (i) a morning sample, acquired at home, within 30 min after awakening and before breakfast; and (ii) an afternoon sample. To assess variations in DNAm of OXTR (hg19, chr3:8,810,729-8,810,845), salivary samples were obtained via the Oragene DNA sample collection kit (DNA Genotek Inc., Canada) at T0, T1, and T2.
Randomization	Nasal spray randomization (permuted-block randomization) was performed by the pharmacy of Heidelberg University Hospital (Germany).
Blinding	Nasal spray blinding was performed by the pharmacy of Heidelberg University Hospital (Germany).

Reporting for specific materials, systems and methods

We require information from authors about some types of materials, experimental systems and methods used in many studies. Here, indicate whether each material, system or method listed is relevant to your study. If you are not sure if a list item applies to your research, read the appropriate section before selecting a response.

Materials & experimental systems

n/a	Involved in the study
☒	☐ Antibodies
☒	☐ Eukaryotic cell lines
☒	☐ Palaeontology and archaeology
☒	☐ Animals and other organisms
☐	☒ Clinical data
☒	☐ Dual use research of concern
☒	☐ Plants

Methods

n/a	Involved in the study
☒	☐ ChIP-seq
☒	☐ Flow cytometry
☒	☐ MRI-based neuroimaging

Clinical data

Policy information about clinical studies

All manuscripts should comply with the ICMJE guidelines for publication of clinical research and a completed CONSORT checklist must be included with all submissions.

Clinical trial registration

the European Clinical Trial Registry number: EudraCT 2018-000769-35

Study protocol

the study protocol can be found in he European Clinical Trial Registry (EudraCT 2018-000769-35)

Data collection

Children with a formal diagnosis of ASD were recruited through the Leuven Autism Expertise Centre of Universitary Hospital Leuven (Belgium), and data collection ran continuously between July 2019 and January 2021. Oxytocin levels were assessed via saliva samples acquired at each assessment session (T0, T1, T2), at two time points: (i) a morning sample, acquired at home, within 30 min after awakening and before breakfast; and (ii) an afternoon sample. To assess variations in DNAm of OXTR (hg19, chr3:8,810,729-8,810,845), salivary samples were obtained via the Oragene DNA sample collection kit (DNA Genotek Inc., Canada) at T0, T1, and T2. All samples were acquired at the Leuven University hospital only the morning oxytocin samples were acquired at home.

Outcomes

Saliva samples were collected as exploratory, secondary endpoints in the context of a larger protocol including clinical-behavioural (Daniels et al., 2023) and neural (Alaerts et al., 2023; Moerkerke et al., 2023) assessments as primary outcomes, as registered at the European Clinical Trial Registry (EudraCT 2018-000769-35) and the Belgian Federal Agency for Medicines and Health products (see Supplementary methods).

Salivary samples were collected using Salivette cotton swabs (Sarstedt AG & Co., Germany) and analysed using a commercial enzyme immunoassay oxytocin ELISA kit (Enzo Life Sciences, Inc., USA) in accordance with the manufacturer's instructions.

DNAm was assessed at three CpG sites of OXTR that have been shown to be impacted in ASD (i.e., -934, -924 and -914, relative to the translation start site; see (Moerkerke, Bonte, et al., 2021)). More detailed information regarding the DNA collection procedures and analyses are provided in Supplementary methods.
